# Strict control of telomerase activation using Cre-mediated inversion

**DOI:** 10.1186/1472-6750-6-10

**Published:** 2006-02-20

**Authors:** Mark D Ungrin, Lea Harrington

**Affiliations:** 1 Institute of Biomaterials and Biomedical Engineering, University of Toronto, Terrence Donnelly Centre for Cellular and Biomolecular Research, 160 College Street, Toronto, Ontario, M5S 3E1, USA; 2 Department of Medical Biophysics, University of Toronto, Ontario Cancer Institute, and Campbell Family Institute for Breast Cancer Research, 620 University Avenue, Toronto, ON M5G 2C1, USA

## Abstract

**Background:**

Human cells appear exquisitely sensitive to the levels of hTERT expression, the telomerase reverse transcriptase. In primary cells that do not express hTERT, telomeres erode with each successive cell division, leading to the eventual loss of telomere DNA, an induction of a telomere DNA damage response, and the onset of cellular senescence or crisis. In some instances, an average of less than one appropriately spliced *hTERT *transcript per cell appears sufficient to restore telomerase activity and telomere maintenance, and overcome finite replicative capacity.

**Results:**

To underscore this sensitivity, we showed that a widely used system of transcriptional induction involving ecdysone (muristerone) led to sufficient expression of *hTERT *to immortalize human fibroblasts, even in the absence of induction. To permit tightly regulated expression of *hTERT*, or any other gene of interest, we developed a method of transcriptional control using an invertible expression cassette flanked by antiparallel loxP recombination sites. When introduced into human fibroblasts with the *hTERT *cDNA positioned in the opposite orientation relative to a constitutively active promoter, no telomerase activity was detected, and the cell population retained a mortal phenotype. Upon inversion of the *hTERT *cDNA to a transcriptionally competent orientation via the action of Cre recombinase, cells acquired telomerase activity, telomere DNA was replenished, and the population was immortalized. Further, using expression of a fluorescent protein marker, we demonstrated the ability to repeatedly invert specific transcripts between an active and inactive state in an otherwise isogenic cell background.

**Conclusion:**

This binary expression system thus provides a useful genetic means to strictly regulate the expression of a given gene, or to control the expression of at least two different genes in a mutually exclusive manner.

## Background

The observation that DNA polymerases synthesize DNA in a unidirectional manner (5'-3') and do not completely replicate the 5' end of a linear DNA template, led Olovnikov and Watson to independently speculate that linear chromosomes would undergo a gradual loss of terminal DNA with each round of DNA replication [1-3]. Subsequently, telomere DNA was indeed found to erode upon continuous cell culture of human fibroblasts [4,5]. Combined with the observation that human somatic cells have a limited replicative capacity in culture that is inversely proportional to the age of the donor [6], it was proposed that telomere attrition was responsible for this so-called 'Hayflick limit' [4,7]. In accord with this hypothesis, the average telomere length in a primary fibroblast population is a stronger predictor of the number of remaining cell doublings than donor age [4,7,8].

Human telomerase contains two essential subunits, an RNA component, hTR [9], which serves as the template for *de novo *telomere synthesis, and hTERT, the telomerase reverse transcriptase [10-13], which was cloned based on sequence similarity with the telomerase reverse transcriptase subunit identified in ciliates and yeast [14-16]. The first functional evidence that TERT comprised the telomerase core was that wild-type hTERT is necessary for telomerase activity in cell extracts [10], and that recombinant hTR and hTERT are necessary and sufficient to reconstitute telomerase activity *in vitro *and in cells [17-19]. While hTR is expressed in most cells, *hTERT *transcription is often repressed in many normal human cell types, however its expression can be detected in some dividing human cells, and *hTERT *mRNA expression is often increased in cancer cells [reviewed in [20,21]]. Some telomerase-positive cell populations have been estimated to average less than one functional *hTERT *mRNA per cell [22,23].

The ectopic expression of *hTERT *is sufficient to immortalize several cell types [reviewed in [20,21]]. In these cell populations, the immortalization does not necessarily correlate with telomerase activity levels or telomere length; in some cases, immortalization can be achieved despite very short average telomere lengths [24-26]. In addition, unlike human tumor cell lines that possess multiple karyotypic abnormalities, primary cells immortalized via hTERT do not usually display overt chromosome rearrangements [27-30]. However, genetic rearrangements have been documented in hTERT-immortalized epithelial cells or fibroblasts, especially after prolonged periods of culture, suggesting that telomerase expression may be permissive for events that promote cellular transformation [31-36]. Further, not all cells that express telomerase become immortalized, and in these populations ploidy changes can occur, and are apparently resolved with continued culture without resulting in a transformed phenotype [37,38].

In an effort to better understand the reversible and/or emergent properties of cells immortalized with *hTERT*, we developed a system to allow the reversible and well-controlled expression of *hTERT *in a cell line that would allow an isogenic comparison between telomerase-positive and telomerase-negative cell populations. Previous studies have established methods for the introduction [27,39-43], and subsequent removal of *hTERT *cDNA [25,44-46], however no system allowed for efficient and repeated reversion between the two states, nor allowed transcriptional regulation of *hTERT *(as opposed to complete excision of *hTERT*). Biochemical induction of hTERT might be used for this purpose, however this method proved incapable of sufficient repression in the absence of induction. Thus, we adopted a method of reversible transposition of a defined DNA sequence flanked by two loxP sites in opposing orientations, using Cre recombinase.

The Cre recombinase recognizes DNA sequences known as loxP sites, and specifically catalyzes recombination between them. Use of this system to excise and inactivate genes of interest by looping out the DNA between two parallel loxP sites on exposure to regulated Cre expression was first demonstrated in murine cells over a decage ago [47]. The same principle has also been used to activate expression of a gene *in vivo *via excision of a "stop" sequence that otherwise blocked transcription [48]. Upon excision, one loxP site is left in the genome, while the other remains in the excised DNA. Cre expression may be induced virally [49] or in a tissue-specific manner by crossing the mice with an appropriate strain expressing a Cre recombinase transgene under the control of a promoter specific to the tissue of interest [48]. More recently, antiparallel loxP sites have been used to invert a specific sequence in mammalian cells, swapping the expression of one gene for another [50]. Here, we show that human cells can be immortalized in a manner strictly dependent upon Cre-mediated inversion of an integrated hTERT cassette, and that inversion allows the strict and multiply reversible expression of transcriptional reporters.

## Results

### Biochemical regulation of *hTERT *transcription is insufficiently stringent

We explored the use of a commercially available biochemical induction system (see Materials and Methods) where the insect hormone ecdysone, or its analogs muristerone or ponasterone, is used to induce transcription of a gene of interest. In this system, constitutively expressed glucocorticoid receptor and retinoid X receptor form a heterodimer in the presence of the inducing agent, and subsequently bind a tandem array of response elements upstream of the exogenous gene, thereby activating transcription [51]. This approach was considered because, in some systems, the absence of muristerone resulted in a very low or undetectable basal level of expression of the target gene. [52]. However, cells containing inducible *hTERT *were rendered telomerase-positive, and the population became immortalized, even in the absence of induction (data not shown). This finding is consistent with the observation that very low levels of the *TERT *mRNA are sufficient to render a cell telomerase positive [22].

### Development of a genetic switch for reversible gene expression

**Figure 1:**
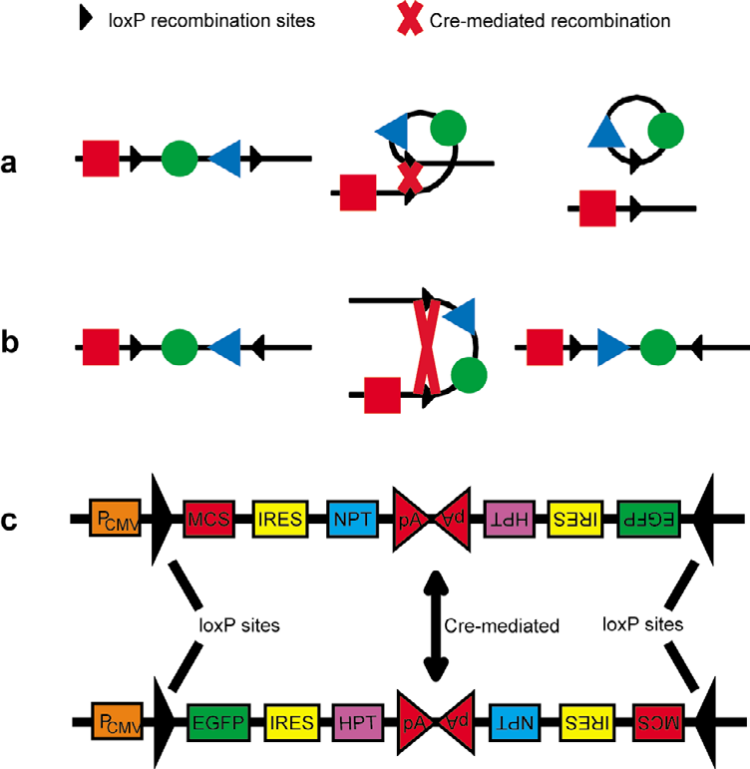
**Schematic of the binary expression vector**. **a**. In one application of the Cre-loxP system, Cre-mediated recombination between two parallel loxP sites results in excision of the intervening sequence. **b**. When two loxP sequences are anti-parallel, Cre-mediated recombination results in inversion of the intervening sequence. **c**. The binary expression vector backbone. Note the antiparallel loxP sites, with a transcriptional promoter upstream of the first loxP site. Other features include drug selectable markers such as NPT (neomycin phosphotransferase) and HPT (hygromycin B phosphotransferase) to allow selection for the desired orientation, and a fluorescence marker, enhanced green fluorescence protein (EGFP). IRES – Internal Ribosome Entry Site, MCS – Multiple Cloning Site, P_CMV _– Cytomegalovirus promoter, pA – polyadenylation signal.

Consequently, we sought a stricter means of regulating gene expression that capitalizes upon the activities of the Cre recombinase (Figure 1) [50,53]. Due to the directionality of Cre-induced recombination, when loxP sites are oriented antiparallel to one another the intervening DNA will be inverted rather than excised (Figure 1). By placing the gene of interest between the loxP sequences, adjacent to transcriptional promoter sequences outside this region, it is possible to juxtapose the gene and the promoter in the same, tandem orientation (to activate transcription), or move them apart and to opposing orientations (thereby deactivating transcription). Further, each orientation can contain a different potential transcript, permitting mutually exclusive regulation of a gene pair. In order to extend the system to bi- or polycistronic gene expression, we also incorporated internal ribosome entry sites (IRES) [54] and genes whose expression would confer drug resistance or fluorescence.

As the two loxP sites are in *cis *(on the same DNA molecule), the inversion reaction is expected to be iterative until Cre expression is lost, as the loxP target sites are not destroyed in the reaction [50,53], thereby generating a racemic population of DNA cassettes in opposite orientations. By virtue of the coupled transcription of the target gene with an antibiotic resistance or fluorescence marker, it was hypothesized that each orientation could be specifically selected from the population. In order to behave as a reversible binary switch, the construct must be present at a single locus; otherwise, multiple copies of the construct could coexist within the same cell in different orientations.

### Multiple inversion of the binary loxP cassette in human cells

**Figure 2:**
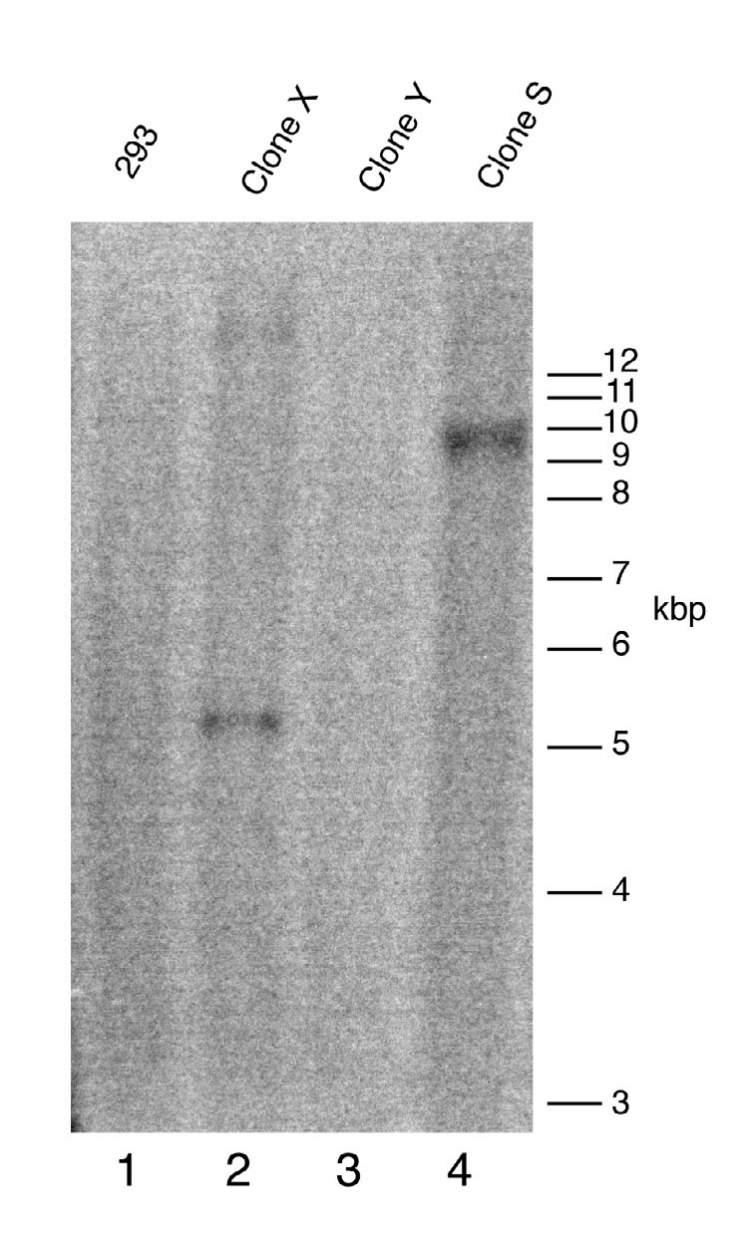
**Isolation of a 293 clone containing a single copy of the binary expression vector**. Genomic DNA (15 μg) prepared from individual 293 cell clones containing an integrated binary expression vector with EGFP in the OFF position, as in Fig 1C. DNA was digested with the restriction enzyme *Xba*I, which cleaves within the construct, approximately 3.5 kbp 3' to the probe sequence (CMV). Thus each individual insertion should result in a single electrophoretic species whose size is dictated by the nearest genomic *Xba*I site. Lane 1, 293 parental cells containing no vector; Lanes 2–3, two random clones (X, Y) that contain more than one cross-hybridizing species; Lane 4, one clone (S), showing a single cross-hybridizing species, as predicted for a single insertion. The positions of the DNA markers are indicated at right, in kilobase pairs (kbp).

**Figure 3:**
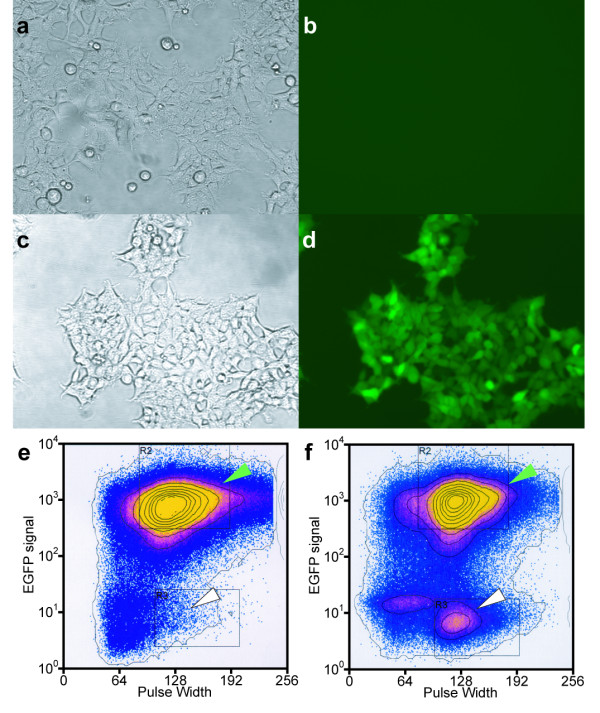
**Multiple switching of the binary expression vector**. **a, b**, the clonal population carrying a single insertion of the binary expression vector (Clone S, Figure 2) by bright field and under fluorescence, respectively. **c, d**, the same population as in panels A and B after introduction of Cre recombinase, and FACS cell sorting to obtain an EGFP-positive population. **e**, FACs analysis of the EGFP-positive population in panel D, with fluorescence plotted logarithmically on the vertical axis and pulse width (particle size) plotted logarithmically on the horizontal axis. **f**, Cre recombinase was introduced into the cell population in panel E, and analyzed by FACS. The major peak at top center (green arrows) represents GFP-positive cells, while the region at lower center is the expected peak location for GFP-negative cells (white arrows). The signal in the lower left quadrant of the FACS profile represents cellular debris. Since the binary vector used to establish Clone S (EGFP OFF) had been switched from ON to OFF *in vitro *prior to introduction into cells (see Materials and Methods), these data establish at least three consecutive switching events are possible.

Initial characterization of the binary vector system was carried out using a version that expressed green fluorescent protein (EGFP) in one orientation but not the other. This binary expression vector, with EGFP in the 'OFF' state (as in Fig. 1C), was introduced into 293 cells via electroporation, cells were plated to low density to select single populations, and Southern blotting of genomic DNA was performed to identify a clonal population that contained a single insertion of the vector DNA, Clone S (Figure 2, lane 4). This population of cells was initially uniformly EGFP negative, as expected (Figure 3, panels A, B). Upon transient introduction of a vector expressing Cre recombinase, a small fraction of the population became stably EGFP positive (data not shown). Cells from this fraction were enriched by fluorescence activated cell sorting (FACS) and plated at low density. Subclones of EGFP positive cells exhibited a stable EGFP positive phenotype over successive cell divisions, and their progeny were uniformly and heritably EGFP positive (Figure 3, panels C and D, and data not shown). Subsequent reintroduction of Cre recombinase, to further iterate the switching process, resulted in the generation of a new population of EGFP negative cells discernible by flow cytometry (Figure 3E, 3F). These data establish that the binary vector system is capable of multiple inversions *in vivo*.

### Application of the binary system to *hTERT*

**Figure 4:**
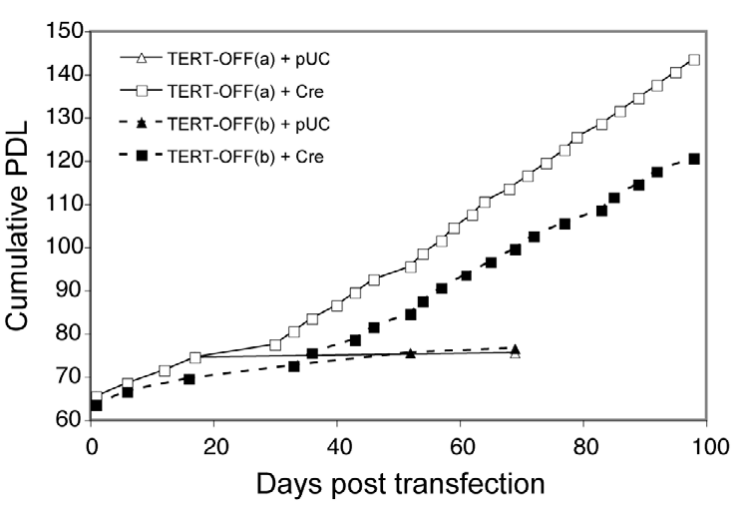
**Strict control of cellular immortalization via the *hTERT *binary vector**. After introduction of a binary expression vector containing *hTERT *in the 'OFF' state, two clonal populations were selected, hTERT-OFF(a) (open symbols) and hTERT-OFF(b) (filled symbols). These distinct cell populations then underwent transient transfection with a control vector, pUC (triangles), or a vector expressing Cre-recombinase (squares). Note that only the cells in which Cre recombinase was introduced became immortalized (squares). The x-axis indicates the number of days, and the y-axis shows cumulative population doublings (PDL).

To determine if the binary system could be used to strictly control *hTERT *expression in human cells, we introduced *hTERT *(followed by puromycin-N-acetyl-transferase) into the binary expression vector, such that *hTERT *was in the 'OFF' position. This vector was introduced into HA-5 cells, which are mortal and derived from human embryonic kidney cells transformed with the SV40 large T and small t antigens [55]. Two clonal populations were selected for further analysis (Figure 4). To one-half of each population was then introduced a control vector (pUC19) or a vector expressing Cre recombinase (pMC-Cre). In the absence of Cre recombinase, the population growth slowed and then arrested, as expected if cells did not express *hTERT *(Figure 4). In contrast to the previous biochemical induction method, cells that contained the *hTERT *cDNA in the 'OFF' position retained a mortal phenotype. Upon Cre expression, however, the cells became immortalized, and continued to divide throughout the duration of the experiment (Figure 4, and data not shown).

### The binary system allows strict control of *hTERT *expression

**Figure 5:**
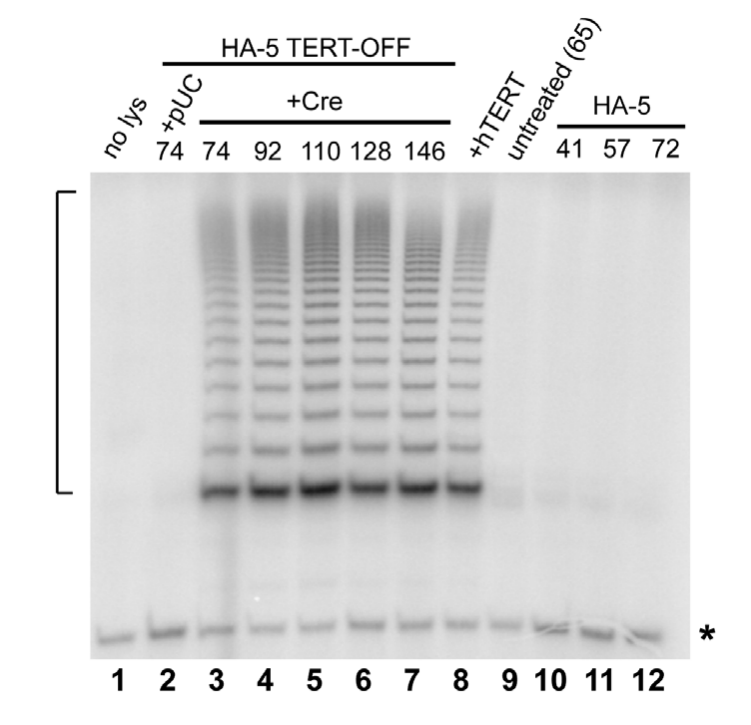
**Reversibility of telomerase activity via the *hTERT *binary vector**. Lysates from hTERT-OFF(a) cells shown in Figure 4 were assayed for telomerase activity by the telomere repeat amplification protocol (TRAP). One microgram total protein was used for each sample, with a 25-cycle amplification step (see Materials and Methods). Lane 1, no lysate added. Lanes 2–7, lysates from HA-5 cells containing the hTERT-OFF vector, followed by introduction of pUC (lane 2) or Cre recombinase (lanes 3–7), at the indicated population doublings. Lane 8, an unrelated population of HA-5 cells containing stably expressed *hTERT*. Lane 9, a population of untreated HA-5 cells split following introduction of hTERT-OFF, at population doubling 65. Lanes 10–12, untreated HA-5 cells, containing no additional vector sequences, at the indicated population doublings. The asterisk indicates a PCR product that serves as an internal positive control for the PCR reaction itself. At left, a bracket shows the amplified telomeric repeats indicative of the presence of telomerase activity.

**Figure 6:**
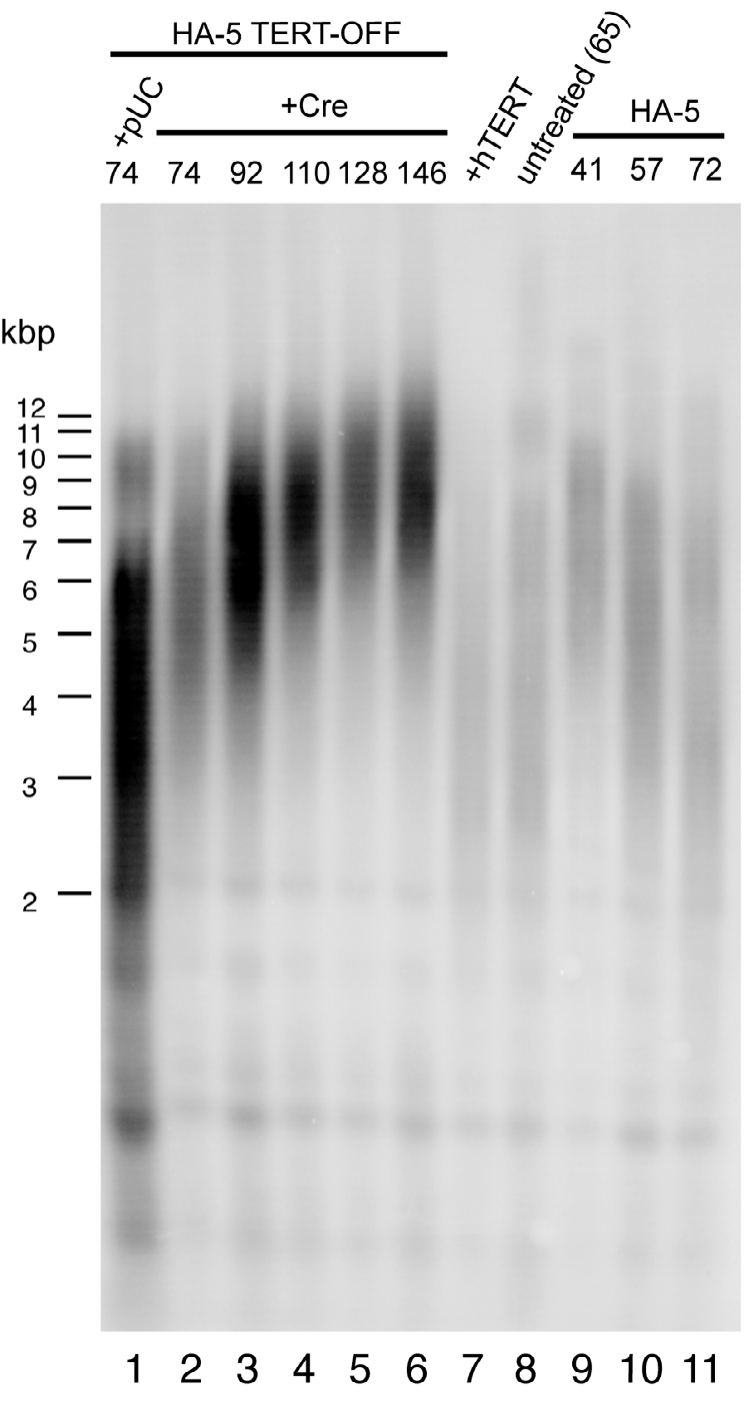
**Reversible telomere length maintenance via the *hTERT *binary vector**. Southern blot analysis of telomere DNA fragments in the same samples shown in Figure 5. Fifteen micrograms of digested genomic DNA was loaded in each lane, and the blot was probed with a radiolabelled telomeric oligonucleotide (see Materials and Methods). The positions of the DNA markers are indicated at left, in kilobase pairs (kbp). Note the gradual elongation of average telomere length in the hTERT-OFF population switched to the 'ON' state (lanes 2–6) relative to HA-5 cells containing no vector (lanes 9–11), or hTERT-OFF cells without addition of Cre (lanes 1, 8).

To determine whether *hTERT *was strictly regulated in the binary system, we examined telomerase activity and telomere length distribution in cell populations containing hTERT in a presumed 'OFF' or 'ON' orientation. In the absence of any vector introduction, HA-5 cells were telomerase negative, as expected (Figure 5, lanes 10–12). Upon introduction of hTERT in the 'OFF' position, cells retained their telomerase-negative status (Figure 5, lanes 2, 8), and became telomerase-positive only upon introduction of Cre recombinase (Figure 5, lanes 3–7). These samples were also analyzed for telomere length distribution by Southern blotting (Figure 6 – see Materials and Methods). Untreated HA-5 cells displayed a gradual attrition in telomere length, as expected (Figure 6, lanes 9–11). A cell population containing *hTERT *in the 'OFF' position showed a mean telomere length similar to untreated HA-5 cells at a similar population doubling (Figure 6, compare lanes 1 and 11). In contrast, in cells in which *hTERT *is flipped to the 'ON' state, the average telomere length is stabilized and even increased upon continuous culture (Figure 6, lanes 2–6). Thus, unlike the biochemical induction method of *hTERT *expression, the binary system described here allows strict control over telomerase activity, and cellular immortalization.

### The use of drug selection via a bi-cistronic transcript

In the binary system, although we were able to successfully and strictly reverse *hTERT *expression, we were unable to select for one vector orientation over another based solely on the drug-resistance of the population (with either G418 or puromycin) when the gene conferring drug resistance was placed after the IRES. We hypothesize this difficulty arose as a consequence of the IRES, from which translation initiation is presumed less efficient. This observation is supported by another study, where the placement of a GFP-encoding cDNA after an IRES element resulted in undetectable GFP fluorescence [56].

## Discussion

Regulable gene function in a manner that is strictly controlled, and iterative, reversible switching between two expression states in an otherwise isogenic background represents a useful and broadly applicable research tool. In the case of introduced *hTERT*, where cells proved to be particularly sensitive to 'leaky' gene expression, we showed that the binary system could successfully switch from a non-immortalized, telomerase-negative population to an immortalized, telomerase-positive population. Moreover, we showed that EGFP fluorescence could be used to successfully select for the multiple inversion of sequences between two loxP sites *in vivo*. While one limitation remains to be overcome, notably the ability to simultaneously select for expression of a second gene following an IRES element, this binary expression system could be extended to any situation in which an integrated expression cassette would benefit from strict control of gene expression. Furthermore, the system possesses the distinct advantage that expression can be efficiently toggled between two genes. As one example, a mutant *hTERT *cDNA (initially in the 'OFF' state) could be placed in the opposite orientation to wild-type *hTERT*, and after selection of immortalized cells containing one copy of the cassette, Cre recombinase-induced switching would allow a comparison of cells containing either wild-type or mutant hTERT for the ability to sustain the immortalized phenotype. In addition, the technique might be further improved by replacing the constitutive transcriptional promoter with an inducible promoter, combining an extremely low background expression when in the 'OFF' state, and the ability to regulate expression levels once the gene has been inverted.

## Conclusion

We have established a binary expression system in which two genes, in opposite orientation and flanked by loxP sites, can be toggled successively between an inactive and active transcriptional state. Using *hTERT *as a sensitive measure of strict gene expression, we showed that cellular immortalization could be achieved only when *hTERT *was placed in the 'active' orientation. This binary expression system promises broad application wherever strict and reversible transcriptional regulation is required.

## Methods

### Construct generation

The binary expression vector was generated using a combination of PCR and standard molecular cloning techniques. The *hTERT *cDNA (Genbank Accession AF015950) was obtained from previously published material [10]. The *EGFP *gene derived from pEGFP-C2 and the IRES and associated antibiotic resistance markers were obtained from pIREShyg, pIRESneo, and pIRESpuro (BD Biosciences, Mississauga, ON). The loxP sequences and one of the polyadenylation sequences were inserted as *de novo *synthesized oligonucleotides. A triple polyadenylation sequence was a kind gift of Dr. Corrine Lobe. Prior to transfection of cells, the binary vector was linearized with the restriction enzyme *Ssp*I, which restricts the DNA outside the CMV promoter and loxP-flanked sequences, to minimize the loss of essential DNA sequences upon integration. Cre recombinase was introduced into cells via the transient transfection of a Cre recombinase expression vector (pMC-Cre, provided by Dr. Razqallah Hakem, Ontario Cancer Institute).

### *In vitro *Cre recombinase switching

*In vitro *Cre recombinase reactions were carried out using the Cre Recombinase kit (Novagen / VWR Canlab, Mississauga, ON) as per the manufacturer's instructions. In brief, after incubation of the binary expression vector with Cre recombinase *in vitro*, the reaction mixture was transformed into *E. coli*, and DNA prepared from single colonies to identify those clones that had undergone inversion between the two loxP sequences.

### Cell culture

Initial characterization of the binary system was carried out in immortal, telomerase-positive 293 cells (Figure 3) [57,58]. The biochemical induction and genetic binary expression of hTERT were carried out in HA-1 and HA-5 cells (kindly provided by Dr. Silvia Bacchetti), respectively, using growth conditions as described in [55].

### Transfection

Transfection of cells with various DNA samples was carried out using the Fugene-6 (Roche, Mississauga, ON), as per the manufacturers instructions.

### Electroporation

Cells were trypsinized followed by addition of PBS with calcium and magnesium, counted and washed in ice-cold PBS without calcium or magnesium and resuspended in ice-cold PBS without calcium or magnesium at 2–5 million cells per mL. One mL was then mixed with 20 μg linearized plasmid DNA and electroporated at 400 V, 250 μF in a Bio-Rad Gene Pulser with Capacitance Extender (Bio-Rad Laboratories, Mississauga, ON).

### Isolation of HA-5 single cell clones

Clonal populations of HA-5 cells were isolated manually, using a pasteur pipet, by removal of adherent colonies visualized under an inverted microscope, followed by placement into a new growth chamber.

### Telomere Repeat Amplification Protocol

TRAP reactions were performed using the TRAPeze kit (Intergen, Purchase, NY, USA) as per the manufacturer's protocol.

### Southern blotting

15 micrograms of each genomic DNA sample, prepared using the DNAzol reagent (Invitrogen, Burlington, ON) following the manufacturer's protocol, was digested with *Xba*I and resolved by electrophoresis in a 0.8% w/v agarose gel. The DNA was then denatured, transferred to Hybond-N+ nylon membrane (Amersham Biosciences, Baie d'Urfé, QC) by capillary methods, and UV crosslinked (240 mJ in a UV Stratalinker 2400, Stratagene, La Jolla, CA, USA). The membrane was probed with radiolabelled DNA probe generated by random priming of a fragment corresponding to the CMV promoter (Figure 2), and washed in 1× SSC, 0.1% w/v SDS at increasing temperature, followed by a final wash in 0.1× SSC, 0.1% w/v SDS. The autoradiographic image was captured using a Storm Phosphorimager (Amersham Biosciences). The Southern blot in Figure 6 was carried out as described above, except that genomic DNA was digested with both *Hin*fI and *Rsa*I, resolved in a 0.5% w/v agarose gel, transferred, and the membrane was probed with a 5' ^32^P-labelled oligonucleotide, (C_3_TA_2_)_3_,.

## Competing interests

The author(s) declare that they have no competing interests.

## Authors' contributions

MU and LH jointly conceived, designed, and interpreted the experiments, and wrote the manuscript. MU performed all experiments with the exception of Figure 3, panels E and F, which were performed by Claude Cantin.
